# The Smaug RNA-Binding Protein Is Essential for microRNA Synthesis During the *Drosophila* Maternal-to-Zygotic Transition

**DOI:** 10.1534/g3.116.034199

**Published:** 2016-09-01

**Authors:** Hua Luo, Xiao Li, Julie M. Claycomb, Howard D. Lipshitz

**Affiliations:** Department of Molecular Genetics, University of Toronto, Ontario M5S 1A8, Canada

**Keywords:** Smaug, miRNA, piRNA, siRNA, RNA-binding protein, RNA degradation, Argonaute, Pumilio, Brain tumor

## Abstract

Metazoan embryos undergo a maternal-to-zygotic transition (MZT) during which maternal gene products are eliminated and the zygotic genome becomes transcriptionally active. During this process, RNA-binding proteins (RBPs) and the microRNA-induced silencing complex (miRISC) target maternal mRNAs for degradation. In *Drosophila*, the Smaug (SMG), Brain tumor (BRAT), and Pumilio (PUM) RBPs bind to and direct the degradation of largely distinct subsets of maternal mRNAs. SMG has also been shown to be required for zygotic synthesis of mRNAs and several members of the *miR-309* family of microRNAs (miRNAs) during the MZT. Here, we have carried out global analysis of small RNAs both in wild-type and in *smg* mutants. Our results show that 85% of all miRNA species encoded by the genome are present during the MZT. Whereas loss of SMG has no detectable effect on Piwi-interacting RNAs (piRNAs) or small interfering RNAs (siRNAs), zygotic production of more than 70 species of miRNAs fails or is delayed in *smg* mutants. SMG is also required for the synthesis and stability of a key miRISC component, Argonaute 1 (AGO1), but plays no role in accumulation of the Argonaute family proteins associated with piRNAs or siRNAs. In *smg* mutants, maternal mRNAs that are predicted targets of the SMG-dependent zygotic miRNAs fail to be cleared. BRAT and PUM share target mRNAs with these miRNAs but not with SMG itself. We hypothesize that SMG controls the MZT, not only through direct targeting of a subset of maternal mRNAs for degradation but, indirectly, through production and function of miRNAs and miRISC, which act together with BRAT and/or PUM to control clearance of a distinct subset of maternal mRNAs.

All animal embryos undergo a maternal-to-zygotic transition (MZT), during which a subset of the maternal gene products that were loaded into the oocyte is eliminated and the zygotic genome becomes transcriptionally active (reviewed in Tadros and Lipshitz 2009; Walser and Lipshitz 2011; Lipshitz 2015). The MZT plays an essential role, permitting handover of developmental control from maternal RNAs and proteins to those encoded by the embryo’s own genome. While the timing and content of the MZT differs between and within phyla, there are several conserved components across the metazoa. First, clearance of a subset of maternal mRNAs occurs in several ‘waves,’ the earliest driven by maternal factors and the later ones dependent on zygotic transcription. Likewise, zygotic genome activation occurs in phases, with an early, minor phase followed by major, large-scale transcription. Another conserved feature of the MZT is the role of RNA-binding proteins (RBPs) and microRNAs (miRNAs), which serve as *trans*-acting specificity factors that recruit the RNA-degradation machinery to their target transcripts.

In *Drosophila*, the MZT occurs very rapidly and is completed within the first several hours of embryogenesis (Blythe and Wieschaus 2015; Harrison and Eisen 2015; Laver *et al.* 2015b). Studies over the past decade have identified several of the factors that direct both maternal mRNA clearance and zygotic genome activation. The first of these to be identified, and the earliest acting factor, is the Smaug (SMG) RNA-binding protein, which is synthesized from maternal mRNA after egg activation, binds to stem-loop structures in its target mRNAs, and recruits the CCR4/NOT-deadenylase complex to destabilize these transcripts (Semotok *et al.* 2005, 2008; Tadros *et al.* 2007; Benoit *et al.* 2009; Siddiqui *et al.* 2012; Chen *et al.* 2014).

During the MZT, SMG binds to hundreds of maternal mRNA species, serving as the specificity factor that triggers their degradation (Chen *et al.* 2014). However, in *smg* mutants, not only these direct targets but hundreds of additional maternal mRNA species fail to undergo clearance (Tadros *et al.* 2007; Benoit *et al.* 2009). We have hypothesized that, at least in part, this is because zygotic genome activation fails in the absence of SMG and, therefore, the transcription-dependent, later waves of maternal mRNA decay are affected indirectly (Benoit *et al.* 2009). Contributing to one of these later waves is the *miR-309* locus, which encodes a dozen species of miRNAs with the capacity to target several hundred maternal transcripts for degradation (Bushati *et al.* 2008). We previously showed that several *miR-309* family miRNA species are not produced in embryos derived from *smg* mutant mothers and that *miR-309*-dependent maternal mRNAs remain stable (Benoit *et al.* 2009).

Here, we have carried out global analyses of small RNAs during the MZT in both wild-type and *smg* mutants. First, by analyzing activated, unfertilized eggs, which are loaded with maternally encoded products but do not undergo zygotic genome activation, we were able to identify maternally loaded small RNAs. Second, by analyzing two embryonic time-points, one during the early phase of maternal transcript clearance before large-scale zygotic genome activation and the second after completion of the both the early phase of clearance and global zygotic genome activation, and comparing these to unfertilized eggs, we were able to identify changes in small-RNA populations during the MZT. Third, by analyzing these same time-points in embryos from *smg* mutant females, we were able to define global roles for SMG in small RNA metabolism during the MZT.

Loss of SMG has a profound effect on miRNAs but little effect on piRNAs or siRNAs. Failure to produce zygotic miRNAs in *smg* mutants correlates with failure to clear maternal mRNAs with seed sequences for these miRNA species. We show that SMG is also required for the accumulation of AGO1 protein during the MZT. Thus, SMG controls the MZT through direct regulation of maternal mRNAs and, indirectly, through miRISC, which targets additional maternal mRNA species for clearance.

Recently, we identified the direct targets of the BRAT and PUM RBPs in early embryos and showed that these targets are distinct from those of SMG (Laver *et al.* 2015a). In addition, we showed that BRAT plays an important role in maternal mRNA decay during the MZT (Laver *et al.* 2015a). Here we show that BRAT and PUM share target mRNAs with those predicted for miRISC during the MZT, and that the *miR-309* family of miRNAs shares predicted targets with BRAT but not PUM. We hypothesize that SMG’s indirect regulation of maternal mRNA clearance is likely to be implemented by miRISC acting together with BRAT and/or PUM.

## Materials and Methods

### Experimental methods

#### Fly stocks:

All *Drosophila* cultures were raised at 25° under standard conditions. The following fly strains were used: (1) *w^1118^*, (2) *VASA-GFP/CyO*; *Pr Dr/TM3*, *Sb*, (3) *VASA-GFP/CyO*; *smg^1^/TM3*, *Sb*, (4) *Df(3L)Scf-R6/TM3*, *Sb* (Bloomington Stock #4500), (5) *smg^47^/TM3* (Chen *et al.* 2014), and (6) *w^1118^*; *P[FLAG.HA.AGO2]2* (Bloomington Stock #33242). We have shown that the *smg^1^* mutation (Dahanukar *et al.* 1999) results in production of a truncated SMG protein lacking the RNA-binding domain (Benoit *et al.* 2009), while the *smg^47^* mutation is a deletion that results in a complete lack of SMG protein (Chen *et al.* 2014). The generation of the SMG^WT^ and the point-mutant SMG^RBD^ transgenic lines has been described previously (Semotok *et al.* 2008). Embryos from *VASA-GFP*; *Pr Dr/TM3*, *Sb* females were used as the wild-type controls and are referred to throughout the text as “wild-type embryos.” VASA-*GFP/CyO*; *smg^1^/Df(3L)Scf-R6* females or *smg^47^* homozygous females were used to produce embryos that are referred to throughout the text as “*smg* mutant embryos” rather than the more cumbersome “embryos produced by *smg* mutant females.”

#### Small RNA library construction and next-generation sequencing:

Samples were collected at the following time-points: 0–2 hr old unfertilized eggs (UF0–2h), 0–2 hr old embryos (F0–2h), and 2–4 hr old embryos (F2–4h). Each time-point had three biological replicates. Total RNA was extracted using TRIzol (Invitrogen) following the manufacturer’s protocol. The small-RNA fraction was isolated from the total RNA sample using a published modification of the mirVana (MirVANA kit, Invitrogen) protocol (Gu *et al.* 2011). 2S rRNA depletion followed a published method (Seitz *et al.* 2008) as did small RNA cloning (Gu *et al.* 2011). High-throughput sequencing of the small RNA libraries was performed using an Illumina HiSeq2500 platform at the Tufts University Core Facility (http://tucf-genomics.tufts.edu/).

#### Western blots:

About 30 μg total protein was resolved by 8% SDS-PAGE. After electrophoresis, proteins were transferred to a PVDF membrane (BioRad, Immun-Blot). The membrane was blocked with 5% milk in TBST (20 mM Tris-Cl, pH 7.5, 150 mM NaCl, and 0.1% Tween20) at room temperature for 1 hr. After blocking, the membrane was incubated overnight at 4° with primary antibody. After washing three times with TBST, the membrane was incubated for 1 hr at room temperature with secondary antibody. Western blots were imaged and quantified using a BioRad Imaging System (ImageLab).

#### Antibodies:

Guinea pig polyclonal anti-SMG antibody (Tadros *et al.* 2007) was used at 1:10,000; guinea pig polyclonal anti-DP1 antibody (Nelson *et al.* 2007) at 1:10,000; mouse monoclonal anti-tubulin antibody at 1:10,000 (Sigma); mouse monoclonal antibody for AGO1 (Miyoshi *et al.* 2005) at 1:1000; and mouse monoclonal anti-FLAG antibody (M2) at 1:1000 (Sigma). Antibodies for Argonautes associated with si- and piRNAs are described in Supplemental Material, File S1.

#### RT-qPCR:

Total RNA was isolated from snap-frozen embryo samples using the TRIzol reagent (Life Technologies). The quantity and quality of the extracted RNA were determined by reading the optical densities at 260 and 280 nm (OD 260 / 280) using a NanoDrop spectrophotometer (Thermo Scientific). For mRNA quantification, 1 µg of total RNA was reverse transcribed in a 20 µl reaction using a Vilo Superscript mix (Life Technologies) following the manufacturer’s protocol. The single-stranded cDNA was used to perform quantitative real-time PCR with the SYBR green PCR master mix (Bio-Rad) using a CFX384 Real-Time System (Bio-Rad). Three technical replicates were performed for each RT primer. Relative levels of different transcripts were determined using the ∆∆CT method. *RpL32* transcript levels were used for normalization. RT-qPCR primers are listed in Table S1 (Tab 5: RT-qPCR primers).

### Computational and bioinformatics methods

#### Small-RNA extraction and annotation:

Prior to read mapping, small-RNA reads were processed using the FASTX-Toolkit (http://hannonlab.cshl.edu/fastx_toolkit/) to demultiplex, remove adaptor sequences, filter sequence quality (phred quality ≥ 20 in 100% of nucleotides), size trim and filter [18–30 nucleotides (nt)], and collapse fastq reads. Filtered reads were mapped to *Drosophila* Genome Release 5.50 using Bowtie 0.12.8 in the -v alignment mode (Langmead *et al.* 2009). We considered only reads with perfect matches to the genome in subsequent analyses except where specifically mentioned. For miRNA expression analysis, we remapped these to a custom-made Bowtie index of reference sequences (see below). Reads mapping to multiple loci were distributed uniformly among these loci. For example, if one read mapped to two locations, each location was assigned 0.5 reads.

Samtools (Li *et al.* 2009) and BEDTools (Quinlan and Hall 2010) were used for data processing and analysis. Representative classes for small RNAs were determined by intersection with the General Feature Format (GFF) file or aligned to a custom-made Bowtie index of reference sequences in the following order: miRNA, small RNA (tRNA, rRNA, snoRNA, snRNA, and ncRNA), *cis*-NAT-loci, transposable element consensus sequence, exon and intron sequence, and intergenic region.

##### miRNA:

Hairpin.fa, mature.fa, and dme.gff3 (version 19) were downloaded from miRBase (Kozomara and Griffiths-Jones 2011). Noncanonical miRNAs, specifically meaning that they were out of range of annotated mature miRNA sequences, were constructed by extending two nt at the 5′-end and five nt at the 3′-end of annotated mature miRNA species.

##### Cis-NAT-siRNA:

We used lists of previously published *cis*-NAT siRNA loci in the ovary (Czech *et al.* 2008; Okamura *et al.* 2008) to extract *cis*-NAT-siRNAs (21 nt only).

##### TE-siRNA and TE-piRNA:

The consensus transposable element sequence (Version 9.4.1 from the Berkeley *Drosophila* Genome Project (http://www.fruitfly.org/p_disrupt/TE.html) was used for transposable element mapping. Previously published genomic piRNA cluster loci (Brennecke *et al.* 2007) were used to check the distribution of both of TE-siRNAs (21 nt only) and TE-piRNAs (≥23 nt) in the genome.

##### Other reference sequences:

tRNA, rRNA, snoRNA, snRNA, ncRNA, and exon, intron, and intragenic regions were downloaded from FlyBase (*Drosophila* Genome Release 5.50).

#### Data normalization and difference expression:

In high-throughput sequencing data, when a small number of highly expressed genes contributes a substantial proportion of the sequenced reads, the remaining genes will falsely appear to be downregulated. For example, the *miR-309* cluster accounts for > 60% of miRNA reads in 2–4 hr wild-type embryo samples. To compensate for this, the trimmed mean of M-values (TMM) normalization method was used for small RNA expression normalization. We used reads per million (RPM) for comparison of expression levels between libraries. Small RNA reads RPM > 1 in at least six of 18 libraries was used as a cutoff. We ran a generalized linear model Likelihood Ratio Test (glmLRT, for multifactor experiments) for our datasets. FDR ≤ 5% was considered significant (edgeR, Robinson *et al.* 2010).

#### Analysis of miRNAs:

To identify nontemplated addition (NTA)-miRNA isoforms, we took the imperfectly matched reads in our small RNA library, trimmed off the last three nucleotides from the 3′-end, and then realigned with the genome reference sequence. A subset of these reads then mapped perfectly to the genome reference; therefore, the mismatch came from the 3′-most three nucleotides. We then realigned these mapped reads to the pre-miRNA sequence without trimming and allowing a maximum of three mismatches. Only the 3′-additional sequences that did not match the pre-miRNA sequence were considered as postcleavage nucleotide modification events. For these three nucleotides, only the following patterns were considered as NTA: match-match-mismatch, match-mismatch-mismatch, and mismatch-mismatch-mismatch.

##### Combined analysis of the three isoform types:

The threshold used was Σ (RPM_canonical_ + RPM_noncanonical_ + RPM_NTA_) ≥ 10 in one or more of the six sample sets (the six sets comprised the three time-points by two genotypes; each set included three biological replicates). See Table 1 and Table S1: Tab 3, Exp noncanonical and NTA miRNAs.

**Table 1:** Expression of isoforms of 154 miRNA species expressed during the MZT

Dataset	Canonical RPM	Noncanonical RPM	3′NTA RPM	Total miRNA RPM	Canonical (%)	Noncanonical (%)	3′NTA (%)
wt.UF0–2h	103,105.0	13,902.3	32,837.7	149,847.0	68.8	9.3	21.9
wt.F0–2h	198,888.9	32,382.9	43,433.6	274,705.4	72.4	11.8	15.8
wt.F2–4h	744,043.2	92,198.8	63,846.7	900,088.6	82.7	10.2	7.1
smg.UF0–2h	184,250.6	19,270.6	42,213.7	245,735.0	75.0	7.8	17.2
smg.F0–2h	74,426.1	9,751.6	33,130.1	117,307.8	63.4	8.3	28.3
smg.F2–4h	165,177.2	30,974.7	42,257.2	238,409.1	69.3	13.0	17.7

RPM, reads per million; 3′NTA, 3′-end nontemplated addition; miRNA, microRNA.

##### Hierarchical clustering of canonical miRNAs (Figure 2):

The threshold for inclusion of a canonical miRNA species was that the mean RPM of that species be ≥10 in at least one of the six sample sets (the six sets comprised the three time-points by two genotypes; each set included three biological replicates). RPM was converted to log_2_ and then miRNA species were classified by hierarchical clustering (*k* = 5) in the R software package (Version 3.1.2) (Shen 2013). Table S1: Tab 2, Expression-profiles of miRNA class.

##### Detailed analysis of a subset of highly expressed noncanonical miRNAs (Figure S2):

To be included in the analysis, a noncanonical miRNA species had to meet three criteria: (1) Σ (RPM_canonical_ + RPM_noncanonical_ + RPM_NTA_) ≥ 10 in one or more of the six sample sets (the six sets comprised the three time-points by two genotypes; each set included three biological replicates); (2) RPM_noncanonical_ / Σ (RPM_canonical_ + RPM_noncanonical_ + RPM_NTA_) ≥ 0.5 in one or more of the six sample sets; and (3) |RPM_wild-type eggs_ − RPM_wild-type embryos_| ≥ 0.2. See Table S1: Tab 3, Exp noncanonical and NTA miRNAs.

##### Detailed analysis of a subset of highly expressed NTA-miRNAs (Figure S2):

To be included in the analysis, an NTA-miRNA species had to meet three criteria: (1) Σ (RPM_canonical_ + RPM_noncanonical_ + RPM_NTA_) ≥ 10 in one or more of the six sample sets (the six sets comprised the three time-points by two genotypes; each set included three biological replicates); (2) RPM_NTA_ / Σ (RPM_canonical_ + RPM_noncanonical_ + RPM_NTA_) ≥ 0.2 in two or more of the six sample sets; and (3) |RPM_wild-type eggs_ − RPM_wild-type embryos_| ≥ 0.1 or |RPM_wild-type embryos_ − RPM*_smg_*_embryos_| ≥ 0.2. See Table S1: Tab 3, Exp noncanonical and NTA miRNAs.

#### Comparative analysis of miRNA targets:

To define the set of canonical miRNAs downregulated in *smg* mutants, we chose canonical miRNAs with FDR ≤ 5% at F0–2h or F2–4h (glmLRT) and RPM_canonical_ ≥ 10 in one or more of the six sample sets (the six sets comprised the three time-points by two genotypes). To identify mRNA targets of these downregulated miRNAs, we downloaded datasets from TargetScanFly 6.2 (Schnall-Levin *et al.* 2010) and extracted *Drosophila melanogaster* conserved target sites in the ORF and 3′UTR. Since TargetScanFly6.2 does not have the seed sequences for all miRNAs, we only chose canonical miRNAs that were in the TargetScanFly list for comparative analysis as described above. Previously defined lists were used for SMG-dependent-for-decay mRNAs (embryos 2–3 hr, 5% FDR) (Benoit *et al.* 2009) and SMG-RIP mRNAs (embryos 0–3 hr, 5% FDR) (Chen *et al.* 2014). Statistical analysis used the one-tailed Fisher’s exact test with the Bioconductor GeneOverlap package (Version 1.6.0) in the R software package (Version 3.1.2) (Shen 2013).

### Data availability

The data reported in this study have been deposited in NCBI’s Gene Expression Omnibus and are accessible through GEO series accession number GSE82194.

## Results and Discussion

### Identification of small RNAs present during the MZT

To identify small RNA species expressed during the *Drosophila* MZT and to assess the role of SMG in their regulation, we produced and sequenced 18 small RNA libraries: nine libraries from eggs or embryos produced by wild-type females and nine from *smg* mutant females (the genotypes of the females are in the *Materials and Methods*). The 18 libraries comprised three biological replicates each from the two genotypes and three time-points: (1) 0–2 hr old unfertilized eggs, in which zygotic transcription does not occur and thus only maternally encoded products are present; (2) 0–2 hr old embryos, the stage prior to large-scale zygotic genome activation; and (3) 2–4 hr old embryos, the stage after large-scale zygotic genome activation. After prealignment processing (see *Materials and Methods*), a total of ∼144 million high quality small-RNA reads was obtained and 110 million of these perfectly matched the annotated *Drosophila* genome (FlyBase 5.50). The components of our small RNA libraries are shown in Table S1 (Tab 1: Small RNA library components).

**Figure 1:**
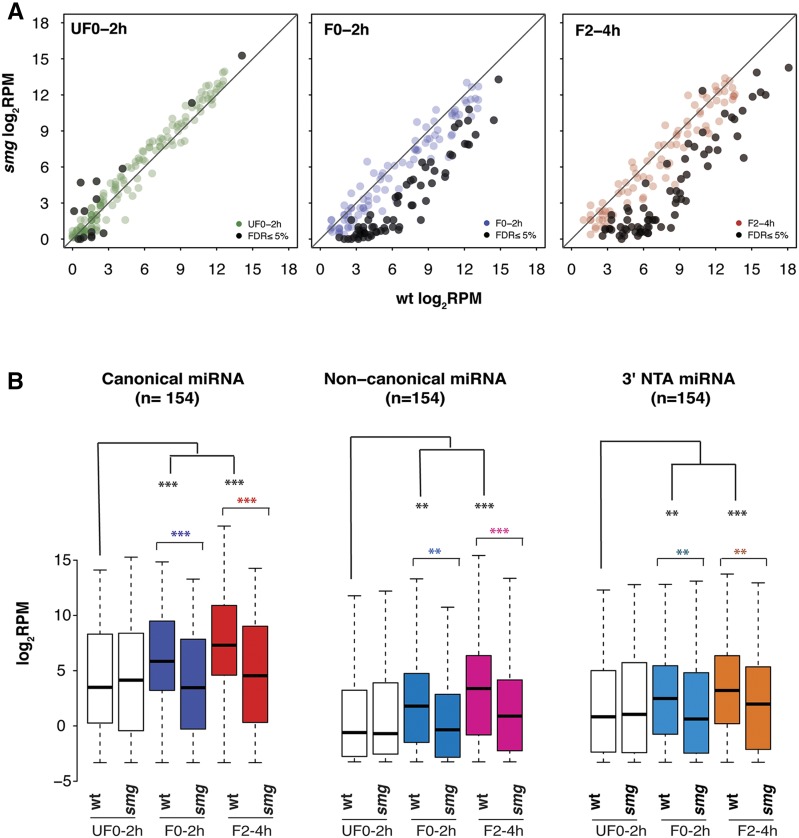
miRNA expression is dramatically reduced in *smg* mutant embryos. (A) Scatterplots showing canonical miRNA expression levels in reads per million (RPM) in wild-type and *smg* mutants in 0–2 hr old unfertilized eggs (UF0–2h), 0–2 hr old embryos (F0–2h), and 2–4 hr old embryos (F2–4h). (B) Box plots showing that canonical, noncanonical, and 3′-end nontemplated addition (NTA) miRNAs were significantly downregulated in *smg* mutant embryos at 0–2 hr and 2–4 hr. *P* values are from the Wilcoxon rank sum test: ** ≤ 0.01, and *** ≤ 0.001. FDR, false discovery rate; miRNA, microRNA; wt, wild type.

**Figure 2:**
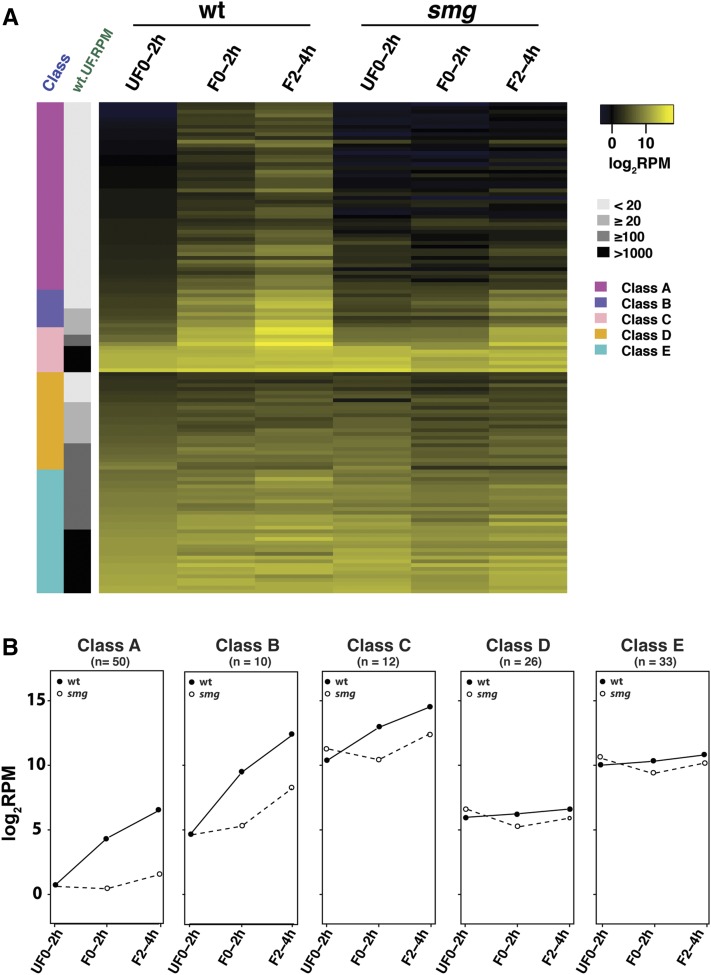
miRNA expression profiles fall into five classes. (A) A heat map showing the expression profiles of each miRNA at the three developmental stages. The miRNA species were classified into five classes (A through E) by hierarchical clustering (*k* = 5). Each row represents one species of miRNA log_2_RPM. The gray bar labeled wt.UF.RPM shows the abundance of maternally loaded miRNAs in RPM as indicated. (B) Plots showing the average expression levels of the miRNA species in each class, in wild-type (solid line) and *smg* mutant (dashed line). UF0-2h, 0-2 hr unfertilized eggs; F0-2h, 0–2 hr embryos; F2-4h, 2–4 hr embryos. miRNA, microRNA; RPM, reads per million; wt, wild type.

**Figure 3:**
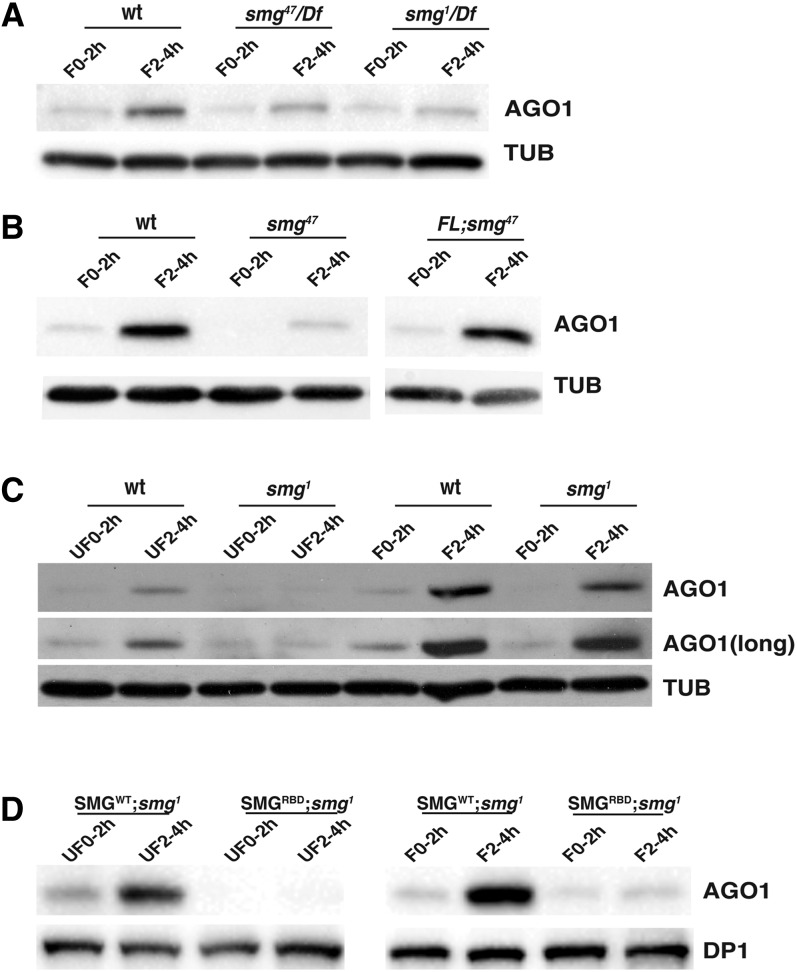
AGO1 protein levels are dramatically reduced in *smg* mutant embryos. Western blot analysis of SMG protein expression with either tubulin or DP1 as loading controls. (A) AGO1 levels in *smg^1^/Df(3L)Scf* and *smg^47^/Df(3L)Scf* embryos are reduced relative to wild-type in 2–4 hr embryos. (B) A full-length *smg* transgene (FL) rescues AGO1 expression in a *smg^47^* mutant background. (C) AGO1 levels are also reduced in *smg^1^/Df(3L)Scf* 2–4 hr unfertilized eggs relative to wild-type. (D) RNA binding by SMG is necessary for AGO1 expression. The genotype of the females was: *UASP[SMG^WT^ or SMG^RBD^]*; *smg^1^*/*P[GAL4*::*VP16-nos.UTR] smg^1^*. The SMG^WT^ transgene rescued expression of AGO1 in both *smg^1^* unfertilized eggs and embryos, whereas the SMG^RBD^ transgene (Semotok *et al.* 2008) did not rescue *smg^1^*. AGO1, Argonaute 1; SMG, Smaug; TUB, tubulin; wt, wild type. UF0-2h, 0-2 hr unfertilized eggs; UF2-4h, 2-4 hr unfertilized eggs; F0-2h, 0-2 hr embryos; F2-4h, 2-4 hr embryos.

**Figure 4:**
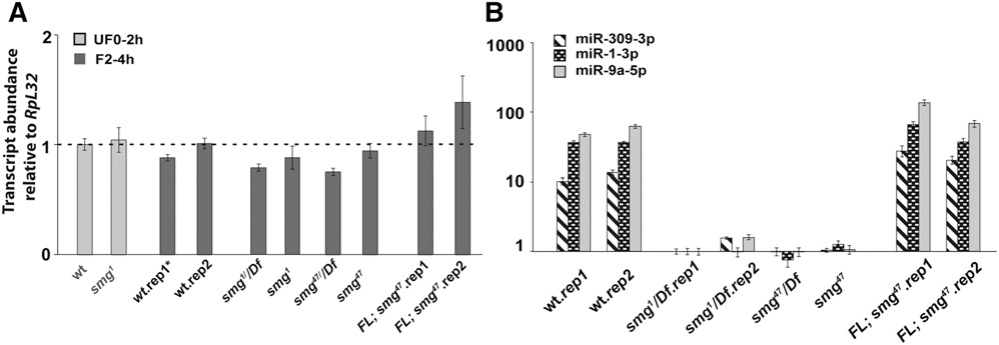
RT-qPCR shows that *Ago1* mRNA expression is unaffected in *smg* mutants whereas miRNA levels are dramatically reduced. (A) RT-qPCR of *Ago1* mRNA shows that expression is unaffected in *smg* mutant eggs and embryos. (B) RT-qPCR shows that a full-length *smg* transgene restores expression of *miR-1-3p*, *miR-9-5p*, and *miR-309-3p* expression in 2–4 hr *smg^47^* mutant embryos. AGO1, Argonaute 1; SMG, Smaug; FL, full-length *smg* transgene; mRNA, messenger RNA; rep, biological replicate; RT-qPCR, reverse transcription-quantitative polymerase chain reaction; wt, wild type.

**Figure 5:**
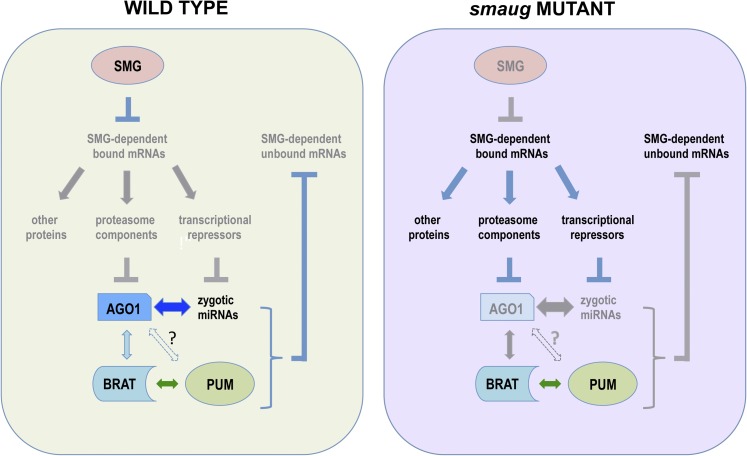
Speculative model for SMG regulation of miRISC in early embryos. Left: In wild type, SMG protein represses mRNAs encoding transcriptional repressors, thus permitting zygotic synthesis of miRNA species, which feed-back to clear additional maternal mRNAs. SMG also represses mRNAs encoding ubiquitylating enzymes and proteasome subunits, thus stabilizing AGO1 protein. The zygotic miRNAs and AGO1 are assembled into miRISC, which further stabilizes these components. BRAT and PUM physically interact with miRISC. Right: In *smg* mutants, the mRNAs encoding transcriptional repressors are not repressed, thus zygotic miRNA species are not produced and their target maternal mRNAs persist. Likewise, the mRNAs related to proteasome function are not repressed, thus AGO1 protein is degraded. The absence of miRNAs results in failure to incorporate AGO1 protein into miRISC, further destabilizing AGO1 protein. In the absence of miRISC, BRAT and PUM are unable to destabilize their mRNA targets. AGO1, Argonaute 1; BRAT, Brain tumor; SMG, Smaug; mRNA, messenger RNA; miRISC, microRNA-induced silencing complex; miRNA, microRNA; PUM, Pumilio.

We found that loss of SMG had no significant effect on piRNAs and siRNAs, or on the Argonaute proteins associated with those small RNAs: Piwi, Aubergine (AUB), AGO3, and AGO2, respectively. In contrast, loss of SMG resulted in a dramatic, global reduction in miRNA populations during the MZT as well as reduced levels of AGO1, the miRISC-associated Argonaute protein in *Drosophila*. Our analyses of miRNAs and AGO1 are presented in the body of this manuscript (Figure 1, Figure 2, Figure 3, Figure 4, Figure 5, Figure S1, Figure S2, Table 1, Table 2, Table 3, and Table S1) while the data on piRNAs, siRNAs, and the AGO2, AGO3, AUB, and Piwi proteins are exclusively in the supplemental data (Figure S3, Figure S4, Figure S5, Figure S6, File S1, and Table S1).

### miRNA isoforms present during the MZT

A pre-miRNA can generate three types of mature miRNA: (1) a canonical miRNA, which has a perfect match to the annotated mature miRNA; (2) a noncanonical miRNA, which shows a perfect match to the annotated mature miRNA but with additional nucleotides at the 5′- or 3′-end that match the adjacent primary miRNA sequence; and (3) a miRNA with nontemplated terminal nucleotide additions (an NTA-miRNA), which has nucleotides at its 3′-end that do not match the primary miRNA sequence.

In our libraries, we identified a total of 364 distinct miRNA species that mapped to miRBase (Version 19), comprising 85% (364/426) of all annotated mature miRNA species in *Drosophila*. Thus, the vast majority of all miRNA species encoded by the *Drosophila* genome are expressed during the MZT. Overall, in the wild type, an average of 75% of all identified miRNAs fell into the canonical category. The remaining miRNAs were either noncanonical (10%) or NTA-miRNAs (15%).

To validate our sequencing results, we compared those mature miRNA species identified in our data that perfectly matched the *Drosophila* genome sequence (*i.e.*, canonical and noncanonical) with a previously published miRNA dataset from 0 to 6 hr old embryos (Ruby *et al.* 2007). To avoid differences caused by miRBase version, we remapped data sets from that previous study (GSM180330 and GSM180331, 0–6 hr) to miRBase Version 19 and found that 99% of their published miRNA species were on our miRNA list (176/178 mature miRNA species comprising 161 canonical miRNAs and 94 noncanonical miRNAs). There were an additional 181 mature miRNA species in our library that had not been identified as expressed in early embryos in the earlier study.

As a second validation, we compared our list of maternally expressed miRNA species (those present in our 0–2 hr wild-type unfertilized egg samples) with the most recently published list of maternal miRNAs, which had been defined in the same manner (Marco 2015). We found that that 99% of the 86 published maternal miRNA species were on our maternal miRNA list (85/86). We identified an additional 144 maternal miRNA species in our library that had not been observed in that study.

Our identification of a large number of additional miRNA species in unfertilized eggs and early embryos can be attributed to the depth of coverage of the current study. Therefore, our dataset provides the most complete portrait to date of the miRNAs present during the *Drosophila* MZT.

### Changes in miRNA populations during the MZT

Next, we analyzed global changes in miRNA species during the MZT in wild-type embryos. We observed a dramatic increase in the proportion of miRNAs relative to other small RNAs, which was due to an increase in absolute miRNA amount rather than a decrease in the amount of other types of small RNAs (Table S1:Tab 1, Small RNA library components). In wild-type 0–2 hr unfertilized eggs, the proportion of our small RNA libraries comprised of canonical and noncanonical miRNAs was 12.8% (mean RPM = 117,068). These represent maternally loaded miRNAs, since unfertilized eggs do not undergo zygotic genome activation. The proportion of small RNAs represented by miRNAs increased dramatically during the MZT, reaching 50.7% in 2–4 hr embryos (mean RPM = 835,638). The other abundant classes of small RNAs underwent either no change or relatively minor changes over the same time-course. We conclude that there is a large amount of zygotic miRNA synthesis during the MZT in wild-type embryos.

For more detailed analysis of the canonical, noncanonical, and NTA isoforms, we focused on 154 miRNA species that possessed an average of ≥10 RPM for all three isoform types in one or more of the six sample sets (the six sample sets comprised three biological replicates each of the two genotypes and three time-points; see Table 1 and *Materials and Methods*). Here, we focus on changes in wild-type; changes in the *smg* mutant will be presented in the next section. Among all miRNAs, in wild-type the proportion of canonical isoforms increased over the time-course from 69 to 83%, the proportion of noncanonical miRNAs remained constant (from 9 to 10%), and the proportion of the NTA-miRNAs decreased (from 22 to 7%). These results derive from the fact that, during the MZT, the vast majority of newly synthesized miRNAs were canonical, undergoing a more than sevenfold increase from 103,105 to 744,043 RPM; that noncanonical miRNAs underwent a comparable, nearly sevenfold, increase from 13,902 to 92,199; whereas NTA-miRNAs underwent a less than twofold increase, from 32,840 to 63,847, thus decreasing in relative proportion.

### Global impact of smg mutations on miRNA populations during the MZT

Whereas the proportion of the small-RNA population that was comprised of miRNAs increased fourfold over the wild-type time-course, concomitant with increases in overall miRNA abundance, there was no such increase in the *smg* mutant embryos: 21.9% of the small RNAs were miRNAs in 0–2 hr unfertilized *smg* mutant eggs (mean RPM = 203,415) and 20.5% (mean RPM = 196,110) were miRNAs in 2–4 hr *smg* mutant embryos (Table S1: Tab 1, Small RNA library components).

This difference between wild-type and *smg* mutants could have resulted from the absence of a small number of extremely highly expressed miRNA species in the mutant. Alternatively, it may have been a consequence of a widespread reduction in the levels of all or most zygotically synthesized miRNAs in *smg* mutants. To assess the cause of this difference, we graphed canonical miRNA reads in scatter plots (Figure 1A). These showed that a large number of miRNA species had significantly reduced expression levels in 0–2 and in 2–4 hr *smg* mutant embryos relative to wild-type. Most of the downregulated miRNA species exhibited a more than fourfold reduction in abundance relative to the wild type (Figure 1A, FDR ≤ 5%; glmLRT). Furthermore, this reduction occurred for miRNA species expressed over a wide (10^4^-fold) range of abundances in wild type.

We then used box plots to analyze the canonical, noncanonical and NTA, isoforms of the 154 miRNA species identified in the previous section (Figure 1B). These showed that, in wild type, the median abundance of canonical, noncanonical, and 3′NTA miRNAs increased significantly in 0–2 and in 2–4 hr embryos relative to 0–2 hr unfertilized eggs. In contrast, there was no significant increase in the median abundance of any of the three isoforms of miRNAs in the *smg* mutant embryos. Also, for all three isoform types, when each time-point was compared between wild-type and *smg* mutant, there was no difference between wild type and mutant in 0–2 hr unfertilized eggs but there was a highly significant difference between the two genotypes at both of the embryo time-points. Whereas the abundance of miRNAs differed between wild-type and mutant embryos, there was no difference in length or first-nucleotide distribution of canonical miRNAs, nor in the nontemplated terminal nucleotides added to NTA-miRNAs (Figure S1).

### Classification of miRNA species into expression classes and the role of SMG

As described above, during the wild-type MZT, canonical miRNAs comprised the major isoform that was present (69–83% of miRNAs). Therefore, we next asked whether miRNA species could be categorized into different classes based on their expression profiles during the wild-type MZT. We analyzed 131 canonical miRNA species that had ≥ 10 mean RPM in at least one of the six datasets (see *Materials and Methods*). Hierarchical clustering of their log_2_RPM values identified five distinct categories of canonical miRNA species during the MZT. We then assessed the effects of *smg* mutations on each of these classes. The data are shown in Figure 2 and Table S1 (Tab 2, Exp-profiles of miR class).

#### Class A (50 miRNA species): Nonmaternal, low zygotic:

##### Wild type:

Maternal deposition of this set of miRNA species was absent or very low with an average RPM of 1.4 in 0–2 hr unfertilized eggs. These species accumulated at low levels upon zygotic genome activation, reaching an average of 72.6 RPM in 2–4 hr embryos. Consistent with our conclusion that this class of miRNAs is zygotically synthesized is the fact that 13 of 21 Class A miRNA species that had been examined in a previous study were shown there to be downregulated when the zygotic transcription factor, Zelda (ZLD), is mutated (Fu *et al.* 2014). Three of the Class A miRNA species are part of the 12-member *miR-309* family, which is expressed zygotically and targets a subset of maternal mRNAs for degradation (Bushati *et al.* 2008). 

##### smg mutants:

Class A miRNAs failed to accumulate in *smg* mutants with an average RPM of 1.2 in 0–2 hr unfertilized eggs, 1.1 RPM in 0–2 hr embryos, and 2.1 RPM in 2–4 hr embryos. Consistent with these observations, we previously showed that one of the Class A species, a *miR-309* family member called *miR-3-5p*, fails to increase in abundance in *smg* mutants (Benoit *et al.* 2009).

#### Class B (10 miRNA species; low maternal, high zygotic):

##### Wild type:

Class B miRNA species were loaded at low levels maternally, averaging 23.7 RPM in 0–2 hr unfertilized eggs, then increasing to an average abundance of 702 RPM in 0–2 hr embryos, and 5557 RPM in 2–4 hr embryos. Nine of 10 Class B miRNA species have been shown to be downregulated in *zld* mutants (Fu *et al.* 2014). Half of the members of this class derive from the *miR-309* family. 

##### smg mutants:

In contrast to the wild type, in *smg* mutants there was no significant increase in average abundance of Class B miRNA species between 0–2 hr unfertilized eggs (23.9 RPM) and 0–2 hr embryos (38.9 RPM). However, their average abundance increased almost 10-fold by 2–4 hr to 320 RPM. These results suggest that, in *smg* mutants, synthesis of Class B miRNA species is delayed or reduced rather than absent. This unexpected conclusion was enabled by the global nature of the current analysis relative to our previous study of a small number of *miR-309* family miRNAs (Benoit *et al.* 2009).

#### Class C (12 miRNA species; high maternal, very high zygotic):

##### Wild type:

Class C miRNA species were found to be maternally loaded at high levels as well as synthesized at very high levels zygotically. They were present in 0–2 hr unfertilized eggs at 1301 RPM average. and increased to averages of 7821 RPM at 0–2 hr and 24,042 RPM at 2–4 hr. Five of the 12 members of this class derive from the *miR-309* family. 

##### smg mutants:

Class C miRNA species reached a roughly similar abundance in 2–4 hr mutant embryos (5752 RPM average) to that seen at 0–2 hr in the wild type (7820 RPM average), suggesting that, as was the case for Class B miRNAs, zygotic production of Class C miRNA species is delayed or reduced but not absent in the mutant. One of the *miR-309* family miRNA species that we previously analyzed by northern blots in *smg* mutants (Benoit *et al.* 2009), *miR-3*, belongs to this class.

#### Class D (26 miRNA species; low maternal, constant level):

##### Wild type:

There was no significant change in the abundance of Class D miRNA species in the embryo time-course relative to unfertilized eggs. They were present in unfertilized eggs at an average of 46.1 RPM that increased to 71.9 by 2–4 hr. Therefore, Class D represents either stable maternal miRNAs with a small amount of zygotic synthesis or a balance in which synthesis of new miRNAs in embryos compensates for turnover of maternal copies. 

##### smg mutants:

The expression profiles of Class D miRNA species were very similar in *smg* mutants and the wild type. We observed a drop in average levels from 73.9 RPM in unfertilized eggs to 28.1 in 0–2 hr mutant embryos followed by an increase to 47.8 in 2–4 hr embryos. These data are consistent with decay of maternally contributed copies together with delayed (or lower level) synthesis of zygotic copies in *smg* mutants relative to the wild type. The small magnitude of these changes did not, however, permit a firm conclusion.

#### Class E (33 miRNA species; high maternal, constant level):

##### Wild type:

Class E miRNA species were present in unfertilized eggs at an average RPM of 1031 and increased only slightly, to 1269 RPM, in 0–2 hr embryos, and then to 1798 RPM in 2–4 hr embryos. Thus, this class comprises stable maternal copies with some new synthesis in embryos or unstable maternal copies with compensatory synthesis in embryos. Consistent with the latter possibility, nine of 22 Class E miRNA species have been shown to be downregulated in *zld* mutants (Fu *et al.* 2014). 

##### smg mutants:

The expression profiles of Class E miRNA species were very similar in *smg* mutants to those seen for Class D. There was a drop in average levels from 1502 RPM in unfertilized eggs to 659 RPM in 0–2 hr mutant embryos, followed by an increase to 1166 RPM in 2–4 hr embryos. This is consistent with the decay of maternally contributed miRNA species compensated for with zygotic transcripts in wild-type but delayed or reduced zygotic expression in the mutant.

In summary, during the MZT, canonical miRNA species could be divided into three abundance categories with respect to maternal contribution: absent (Class A; average RPM 1.5), low (Classes B and D; average RPM < 100), or high (Classes C and E; average RPM > 1000). They fell into two categories with respect to their expression profiles during the MZT: significant increase in abundance (Classes A, B, and C) or relatively constant abundance (Classes D and E), the latter likely due to decay of maternal copies compensated for by zygotic synthesis. In *smg* mutants, there was a major deficit in production of zygotic miRNA species, with a striking impact on Classes A, B, and C, but only a minor effect on Classes D and E. Unexpectedly, our data showed that, in *smg* mutants, zygotic synthesis of miRNAs is not absent as was previously thought but is, instead, delayed or occurs at reduced levels. It is not possible to extend analysis of miRNA populations beyond the 2–4 hr time-point in the *smg* mutant since mutant embryos fail to cellularize and then undergo catastrophic death (Benoit *et al.* 2009).

### Impact of smg mutations on expression profiles of noncanonical and NTA-miRNAs during the MZT

For more detailed analysis of a subset of the noncanonical and NTA isoforms in both wild-type and *smg* mutants, we focused on miRNA species for which the noncanonical or NTA isoform represented a substantial proportion of that miRNA’s isoforms and that underwent dynamic changes during the MZT (for details, see *Materials and Methods*).

#### Noncanonical miRNAs:

Consistent with published observations (Fernandez-Valverde *et al.* 2010; Berezikov *et al.* 2011), most of the noncanonical miRNAs in our dataset exhibited the 3′-extended version. The noncanonical isoforms of 10 miRNAs met the above-specified criteria. Strikingly, six of these increased in relative proportion and abundance during the wild-type MZT but showed little or no increase in either relative proportion or absolute abundance in *smg* mutants (Figure S2 and Table S1: Tab 3, Exp noncanonical and NTA miRNAs). These data are consistent with the hypotheses that, for these miRNA species, (1) there is a higher relative production of the noncanonical than the canonical isoform during the MZT in the wild type and (2) there is a failure (or delay) in production of new canonical and noncanonical isoforms in *smg* mutants.

#### NTA-miRNAs:

Recently, it has been shown that maternally loaded miRNAs are highly adenylated (Lee *et al.* 2014). Consistent with this, we observed a higher proportion of NTA miRNAs in our wild-type unfertilized egg samples than in our embryo samples (Table 1). Most of the nontemplated terminal modifications detected were mono- or di-nucleotide additions at the 3′-end (Zhou *et al.* 2012), which are known to influence miRNA stability and target mRNA regulation (Katoh *et al.* 2009). Adenine has been shown to be the most frequent nontemplated 3′-nucleotide (Burroughs *et al.* 2010; Fernandez-Valverde *et al.* 2010; Lee *et al.* 2014); we found that A-addition occurred for a large percentage of the NTA-miRNAs: A (48–50%), AA (12–20%), U (7–12%), AAA (2–8%), C (3–6%), and G (1–2%) (Figure S1).

The NTA isoforms of 25 miRNAs met the above-mentioned criteria, 10 of which showed particularly striking changes during the MZT in wild-type but not in *smg* mutants (Figure S2 and Table S1: Tab 3, Exp noncanonical and NTA miRNAs). Whereas their canonical versions all underwent increases in both proportion and abundance during the MZT, all 10 NTA isoforms underwent an increase in abundance but a decrease in relative proportion during the wild-type MZT. This is likely to be a consequence of production of canonical and noncanonical isoforms outstripping production of NTA isoforms during the MZT. In contrast, in *smg* mutants, all 10 NTA miRNA isoforms showed either no absolute increase in abundance or a very small increase when compared to the wild type. However, relative to the wild type, in the mutant they showed either a smaller relative decrease in proportion or even an increase in proportion. These results are consistent with replacement of maternally loaded NTA-miRNAs with the zygotically produced canonical or noncanonical counterparts during the MZT. Because of the observed decrease in zygotic synthesis of canonical and noncanonical isoforms in *smg* mutants, the amplitude of the relative decrease was smaller (or there was a relative increase) for the NTA isoform.

### Indirect clearance of maternal mRNAs by SMG-dependent miRNAs

We previously identified SMG-bound mRNAs and showed that the vast majority of these depend on SMG for clearance during the MZT (Benoit *et al.* 2009; Chen *et al.* 2014). However, many maternal mRNAs that depend on SMG for clearance are not bound by SMG, suggesting indirect regulation. With respect to this latter possibility, the current study permitted us to assess on a global scale whether maternal mRNAs that are SMG-bound and/or SMG-dependent for degradation are enriched for predicted target sites for miRNAs that are downregulated in *smg* mutants. To do so, we extracted from TargetScanFly 6.2 (Schnall-Levin *et al.* 2010) a list of mRNAs expressed maternally and possessing conserved target sites for those miRNA species with significantly reduced expression in *smg* mutant embryos (see *Materials and Methods* and Table S1, Tab 4). We then compared this list to SMG’s direct targets in 0–3 hr embryos (Chen *et al.* 2014) and the maternal mRNAs dependent on SMG for degradation during the MZT (Benoit *et al.* 2009).

The results of our analysis are shown in Table 2. There was no significant overlap of the list of predicted mRNA targets of SMG-dependent miRNAs with the list of SMG’s direct targets. However, there was a highly significant overlap of the predicted mRNA targets of SMG-dependent miRNAs with those mRNAs that depend on SMG for degradation. When we removed SMG’s direct targets from the latter list, keeping only indirect targets, the overlap increased in significance.

**Table 2:** Overlap of mRNAs that are bound or regulated by Smaug, and those that are targets of miRNAs that are downregulated in *smaug* mutants

Dataset	Number	Overlap	*P* Value*^a^*	Odds Ratio
Expressed genes for Smaug-dependent decay (embryos 2–3 hr)	3956			
Smaug-dependent decay genes (embryos 2–3 hr, 5% FDR)*^b^*	418			
Smaug-dependent-miR target genes	1507	151	4.9 × 10^−6^	1.6
Expressed genes for Smaug-dependent decay (embryos 2–3 hr)	3956			
Smaug-dependent decay genes (embryos 2–3 hr, 5% FDR)*^c^*	341			
Smaug-dependent-miR target genes	1507	128	3.1 × 10^−6^	1.7
Expressed genes for Smaug-RIP (embryos 0–3 hr)	4481			
Smaug-RIP genes (embryos 0–3 hr, 5% FDR)	339			
Smaug-dependent-miR target genes	1200	101	0.11	1.1

FDR, false discovery rate; miR, microRNA.

a One-tailed Fisher’s exact test.

b Smaug-dependent decay genes list including Smaug-IP-genes.

c Smaug-dependent decay genes list without Smaug-IP-genes.

These data are consistent with a model in which SMG degrades its direct targets without the assistance of miRNAs, whereas a large fraction of the indirectly affected maternal mRNAs in *smg* mutants fails to be degraded by virtue of being targets of zygotically produced miRNA species that are either absent or present at significantly reduced levels in *smg* mutants. Thus, SMG is required both for early, maternally encoded decay and for late, zygotically encoded decay. In the former case, SMG is a key specificity component that directly binds to maternal mRNAs; in the latter case, SMG is required for the production of the miRNAs (and AGO1 protein, see below) that are responsible for the clearance of an additional subset of maternal mRNAs (this model is schematized in Figure 5).

### SMG is required for AGO1 accumulation during the MZT

In *Drosophila*, the stability of miRNAs is enhanced by AGO1 and vice versa (Smibert *et al.* 2013). Since miRNA levels are dramatically reduced in *smg* mutants, we decided to assess *Ago1* mRNA and AGO1 protein levels during the MZT both in the wild type and in *smg* mutants. In the wild type, AGO1 levels were low in unfertilized eggs and 0–2 hr embryos but then increased substantially in 2–4 hr embryos (Figure 3). These Western blot data are consistent with an earlier, proteomic study that reported a more than threefold increase in AGO1 in embryos between 0–1.5 hr and 3–4.5 hr (Gouw *et al.* 2009). In contrast to AGO1 protein, we found that *Ago1* mRNA levels remained constant during the MZT (Figure 4). Taken together with a previous report that *Ago1* mRNA is maternally loaded (Lott *et al.* 2011), the increase in AGO1 protein levels in the embryo is, therefore, most likely to derive from translation of maternal *Ago1* mRNA rather than from newly transcribed *Ago1* mRNA.

Next, we analyzed AGO1, AGO2, AGO3, AUB, and Piwi protein levels in eggs and embryos from mothers carrying either of two *smg* mutant alleles: *smg^1^* and *smg^47^* (for details of the molecular lesions see *Materials and Methods*; Benoit *et al.* 2009; Chen *et al.* 2014). The *smg* mutations had no effect on the expression profiles of AGO2, AGO3, AUB, or Piwi (Figure S5). In contrast, in *smg* mutant embryos, the amount of AGO1 protein at both 0–2 and 2–4 hr was reduced relative to the wild type, and this defect was rescued in embryos that expressed full-length, wild-type SMG from a transgene driven by endogenous *smg* regulatory sequences (Chen *et al.* 2014) (Figure 3, A and B). The reduction of AGO1 protein levels in *smg* mutants was not a secondary consequence of reduced *Ago1* mRNA levels, since *Ago1* mRNA levels in both the *smg* mutant and the rescued-*smg* mutant embryos were very similar to the wild type (Figure 4).

As mentioned above, a plausible explanation for the decrease in AGO1 levels in *smg* mutants is the reduced levels of miRNAs, which would then result in less incorporation of newly synthesized AGO1 into functional miRISC and consequent failure to stabilize the AGO1 protein (Smibert *et al.* 2013). To assess this possibility, we analyzed a time-course in wild-type unfertilized eggs in which zygotic genome activation and, therefore, zygotic miRNA synthesis, does not occur. We found that AGO1 levels were reduced in 2–4 hr wild-type unfertilized eggs compared with wild-type embryos of the same age (Figure 3C). This result is consistent with a requirement for zygotic miRNAs in the stabilization of AGO1 protein.

We next compared wild-type unfertilized egg and *smg* mutant unfertilized egg time-courses, and found that AGO1 levels were further reduced in the *smg* mutant relative to the wild type (Figure 3C). This suggests that SMG protein has an additional function in the increase in AGO1 protein levels that is independent of SMG’s role in zygotic miRNA production (since these are produced in neither wild-type nor *smg* mutant unfertilized eggs).

To assess whether this additional function derives from SMG’s role as a posttranscriptional regulator of mRNA, we rescued *smg^1^* mutants either with a wild-type SMG transgene driven by the Gal4:UAS system (SMG^WT^) or a GAL4:UAS-driven transgene encoding a version of SMG with a single amino acid change that abrogates RNA binding (SMG^RBD^) and, therefore, is unable to carry out posttranscriptional regulation of maternal mRNAs (Semotok *et al.* 2008). We found that, whereas AGO1 was detectable in both unfertilized eggs and embryos from SMG^WT^-rescued mothers, AGO1 was undetectable in unfertilized eggs from SMG^RBD^-rescued mothers and was barely detectable in embryos from these mothers (Figure 3D). Thus, SMG’s RNA-binding ability is essential for its non-miRNA-mediated role in regulation of AGO1 levels during the MZT.

Since the abundance of SMG^WT^ and SMG^RBD^ proteins is very similar (Semotok *et al.* 2008), the preceding result excludes the possibility that it is physical interaction between SMG and AGO1 (Pinder and Smibert 2013) that stabilizes the AGO1 protein. We have previously shown that the *Ago1* mRNA is not bound by SMG (Chen *et al.* 2014). Thus, SMG must regulate one or more other mRNAs whose protein products, in turn, affect the synthesis and/or stability of AGO1 protein. It is known that turnover of AGO1 protein requires Ubiquitin-activating enzyme 1 (UBA1) and is carried out by the proteasome (Smibert *et al.* 2013). We have previously shown that the *Uba1* mRNA is degraded during the MZT in a SMG-dependent manner, and that both the stability and translation of mRNAs encoding 19S proteasome regulatory subunits are upregulated in *smg* mutant embryos (Chen *et al.* 2014). We speculate that increases in UBA1 and proteasome subunit levels in *smg* mutants contribute to a higher rate of AGO1 turnover and, thus, lower AGO1 abundance than in the wild type (schematized in Figure 5).

### The BRAT and PUM RBPs may cooperate with miRISC in maternal mRNA clearance

*Drosophila* AGO1 physically associates with BRAT (Neumuller *et al.* 2008). It is not known whether AGO1 interacts with PUM, but it has been reported that, in mammals and *C. elegans*, Argonaute family proteins interact with PUM/PUF family proteins (Friend *et al.* 2012). We recently identified direct target mRNAs of the BRAT and PUM RBPs in early *Drosophila* embryos and showed, through analysis of *brat* mutants, that during the MZT, BRAT directs late (*i.e.*, after zygotic genome activation) decay of a subset of maternal mRNAs (Laver *et al.* 2015a). These data permitted us to ask whether the maternal mRNAs that are predicted to be indirectly regulated by SMG via its role in miRISC production might be coregulated by BRAT and/or PUM.

We found a highly significant overlap between the predicted miRNA-dependent indirect targets of SMG and both BRAT- and PUM-bound mRNAs in early embryos (Table 3). This suggests that BRAT and PUM might function together with miRISC during the MZT to direct decay of maternal mRNAs (schematized in Figure 5).

**Table 3:** Overlap of mRNAs that are bound by BRAT and PUM and those that are stabilized in a *miR-309* family deletion mutant

Dataset	Number	Overlap	*P* Value*^a^*	Odds Ratio
Expressed genes for BRAT and PUM RIP (0–3 hr)	6191			
BRAT-RIP genes (0–3 hr, 5% FDR)	1197	425	1.1 × 10^−15^	1.75
PUM-RIP genes (0–3 hr, 5% FDR)	641	265	7.4 × 10^−19^	2.18
miRNA-target genes without Smaug-IP-genes	1622			
Expressed genes for BRAT and PUM RIP (0–3 hr)	6191			
BRAT-RIP genes (0–3 hr, 5% FDR)	1197	107	1.1 × 10^−6^	1.82
PUM-RIP genes (0–3 hr, 5% FDR)	641	37	0.57	0.98
*miR-309* cluster-dependent unstable mRNAs (2–3 hr)	363			

BRAT, Brain tumor; PUM, Pumilio; FDR, false discovery rate; miRNA, microRNA.

a One-tailed Fisher’s exact test.

Given that BRAT and PUM bind to largely nonoverlapping sets of mRNAs during the MZT (Laver *et al.* 2015a), there are three types of hypothetical BRAT-PUM-miRISC-containing complexes: one with both BRAT and PUM, one with BRAT only, and one with PUM only. To assess this possibility for a specific set of zygotically produced miRNAs, we compared the lists of mRNAs stabilized in 2–3 hr old embryos from *miR-309* deletion mutants (Bushati *et al.* 2008) to the lists of BRAT and PUM direct-target mRNAs (Laver *et al.* 2015a). The results are shown in Table 3. There was no significant overlap of PUM-bound mRNAs with those upregulated in *miR-309* mutants. However, there was a highly significant overlap of mRNAs upregulated in *miR-309*-mutant embryos with BRAT-bound mRNAs. These results lead to the hypothesis that BRAT (but not PUM) coregulates clearance of *miR-309* family miRNA target maternal mRNAs during the MZT. Experimental tests of this hypothesis are beyond the scope of the present study.

## 

## Supplementary Material

Supplemental Material
