# Supplementary material for: The Smaug RNA-Binding Protein Is Essential for microRNA Synthesis During the Drosophila Maternal-to-Zygotic Transition
Source: G3 (Bethesda). 2016 Sep 1;6(11):3541–51. doi: 10.1534/g3.116.034199 (PMC5100853; doi:10.1534/g3.116.034199)
Supplement: Supplemental Material [file supp_6_11_3541__index.html]

The Smaug RNA-Binding Protein Is Essential for microRNA Synthesis During the Drosophila Maternal-to-Zygotic Transition — Supplemental Material 

# The Smaug RNA-Binding Protein Is Essential for microRNA Synthesis During the *Drosophila* Maternal-to-Zygotic Transition

## Supplemental Material for Luo *et al.*, 2016

**Files in this Data Supplement:**

- File S1 - Analysis of piRNAs and siRNAs. (.pdf, 127 KB)
- Figure S1 - miRNA expression profiles in wild-type and smg-mutant embryos. (.pdf, 630 KB)
- Figure S2 - Expression profiles of specific non-canonical or NTA miRNAs in wild-type and *smg*-mutant embryos. (.pdf, 621 KB)
- Figure S3 - TE-piRNA and TE-siRNA expression in wild-type and *smg*-mutant embryos. (.pdf, 927 KB)
- Figure S4 - piRNA ping-pong signatures, sense-antisense overlap and first-nucleotide distributions in wild-type and *smg*-mutant embryos. (.pdf, 621 KB)
- Figure S5 - Expression of piRNAs and siRNAs from two piRNA cluster loci. (.pdf, 795 KB)
- Figure S6 - Expression of Piwi, AUB, AGO-2 and AGO-3 proteins in wild type and *smg* mutants. (.pdf, 987 KB)
- Table S1 - Small RNA library components. (.xlsx, 113 KB)
