# Supplementary material for: The Smaug RNA-Binding Protein Is Essential for microRNA Synthesis During the Drosophila Maternal-to-Zygotic Transition
Source: G3 (Bethesda). 2016 Sep 1;6(11):3541–51. doi: 10.1534/g3.116.034199 (PMC5100853; doi:10.1534/g3.116.034199)
Supplement: Supplemental Material [file supp_g3.116.034199_FileS1.pdf]

Supplementary text S1.
Analysis of piRNAs and siRNAs

Materials and Methods

Small-RNA library component annotation

Samtools (LI *et al.* 2009b) and BEDTools (QUINLAN AND HALL 2010) were used for data processing and analysis. Representative classes for small RNAs were determined by intersection with the GFF (General Feature Format) file or aligned to a custom-made Bowtie index of reference sequences in the following order: miRNA, small RNA (tRNA, rRNA, snoRNA, snRNA, ncRNA), cis-NAT-loci, transposable element consensus sequence, exon and intron sequence, intergenic region.

miRNA: hairpin.fa, mature.fa and dme.gff3 (version 19) were downloaded from miRBase (KOZOMARA AND GRIFFITHS-JONES 2011). Non-canonical miRNAs – specifically meaning they were out of range of annotated mature miRNA sequences – were constructed by extending two nucleotides at the 5'-end and five nucleotides at the 3'-end of annotated mature miRNAs.

Cis-NAT-siRNA: We used lists of previously published cis-NAT siRNA loci (CZECH *et al.* 2008; OKAMURA *et al.* 2008) to extract cis-NAT-siRNAs (21-nt only).

TE-siRNA and TE-piRNA: The consensus transposable element sequence (Version 9.4.1 from the Berkeley Drosophila Genome Project, (http://www.fruitfly.org/p_disrupt/TE.html) was used for transposable element mapping. Previously published genomic piRNA cluster loci (BRENNECKE *et al.* 2007) were used to check the distribution of both of TE-siRNAs (21 nt only) and TE-piRNAs (≥ 23 nt) in the genome.

Other reference sequences: tRNA, rRNA, snoRNA, snRNA, ncRNA, exon, intron and intragenic regions were downloaded from FlyBase (*Drosophila* Genome Release 5.50).

piRNA cluster, transposon and ping-pong analysis

Prior to alignment to consensus TE sequence or piRNA cluster analysis, we removed miRNA, NAT-siRNA and other small RNAs (such as rRNA, tRNA, snoRNA, etc.). The remaining small-RNA reads were mapped to the consensus transposable element sequence (Version 9.4.1 from the Berkeley *Drosophila* Genome Project, http://www.fruitfly.org/p_disrupt/TE.html). To account for variations within elements, up to three mismatches, but no internal deletions or extensions, were permitted during alignment. Reads mapping to multiple loci were distributed uniformly among these loci.

For all piRNA cluster analyses, only those small RNAs were considered that mapped exclusively to the indicated clusters. The cluster-mapped piRNAs or siRNAs were normalized to their library TMM factors (see Data normalization and difference expression in the Materials and Methods section of the main text, and Supplementary Table S1 Tab1).

Ping-pong signature analysis measures the likelihood that a piRNA has a 'partner' with a 10nt overlap, as described (BRENNECKE *et al.* 2008).

TE-siRNA and TE-piRNA density

The number of siRNAs or piRNAs that mapped to a particular feature (*e.g.*, a transposon consensus sequence or a piRNA cluster) per unit length, was represented as vertical lines over the length of a feature. Densities were drawn based on whether small

RNAs mapped as sense (above) or antisense (below) products to the feature of interest. Densities of small RNA over TEs were drawn using a window size of 1 nt and a step size of 1 nt. All plots of mapped TE-siRNAs or TE-piRNAs over clusters were performed with a window size of 250 nt and a step size of 25 nt.

Western blots

30 µg of total protein was resolved by 8% SDS-PAGE. After electrophoresis, proteins were transferred to a PVDF membrane (BioRad, Immun-Blot). The membrane was blocked with 5% milk in TBST (20 mM Tris-Cl, pH 7.5, 150 mM NaCl, 0.1% Tween20) at room temperature for 1 hr. After blocking, the membrane was incubated overnight at 4 ºC with primary antibody. After washing three times with TBST, the membrane was incubated 1 hr at room temperature with secondary antibody. Western blots were imaged and quantified using a BioRad Imaging System (ImageLab).

Antibodies

Mouse monoclonal anti-tubulin antibody (Sigma) was used at 1:10,000. Mouse monoclonal antibodies for AGO3, Piwi, AUB (MIYOSHI *et al.* 2005; GUNAWARDANE *et al.* 2007; NISHIDA *et al.* 2007) and M2 anti-FLAG antibody (Sigma) to visualize FLAG-AGO2 were used at 1:1,000 -2000.

Results

To identify both piRNAs and siRNAs we only considered reads that uniquely mapped to the *Drosophila* genome for further re-alignment to a custom-made Bowtie index of

reference sequences. After TMM normalization together with other uniquely mapping small RNAs, reads of length greater than 22nt that mapped to consensus TE sequences or piRNA clusters were considered to be piRNAs, and 21nt reads that mapped to relevant the reference sequences to be siRNAs. To identify piRNAs we aligned those reads to repBase (JURKA *et al.* 2005) and BDGP's defined transposable elements (TEs) (CELNIKER *et al.* 2002) separately. To identify siRNAs we used the published ovary endo-siRNA generating loci and cis-NAT loci (Cis-natural antisense transcripts) (CZECH *et al.* 2008) to extract cis-NAT-siRNAs and consensus TEs (BDGP, repBase) for TE-siRNA mapping. TE-siRNAs were generally expressed at much higher levels than cis-NAT-siRNAs across the all embryo time points.

Global piRNA and siRNA profiles were very similar in wild-type and *smaug*-mutant embryos at both the 0-2 and the 2-4 hr time points (Supplementary Figure S2). We also calculated the total piRNA 'ping-pong' signature. There was no significant difference in ping-pong signal pattern or abundance in *smaug* versus wild type, nor was there a difference in normalized piRNA length distribution, or the 5' first nucleotide distribution of piRNAs (Supplementary Figure S3).

To avoid bias caused by alignment to the consensus TE reference, we tested whether a different reference sequence would get similar results. To do so we analyzed piRNAs derived from specific loci, the 'piRNA clusters' (BRENNECKE *et al.* 2007), and we tested the top clusters that had shown altered piRNA expression in *piwi*, *aub, ago3, spnE*, *Rhino* and *armi* mutants (LI *et al.* 2009a; MALONE *et al.* 2009)). The abundance of TE-siRNAs is much lower than TE-piRNAs. Cluster 1 (42AB) and Cluster 2 (20A) are known to show relatively high siRNA expression in early embryos (0-2 hr) (LAU *et al.*

2009); we therefore were particularly interested in the siRNAs from those clusters.

Cluster-1 (42AB): is a strongly maternally inherited, dual-strand piRNA cluster. Cluster 1 piRNA and TE-siRNA density, length distribution and ping-pong signature were very similar between *smaug* and wild type (Supplementary Figure S4).

Cluster 2 (20A): is a strong maternally inherited, uni-strand piRNA cluster. Cluster 2 piRNA and TE-siRNA abundance and length were very similar in *smaug* mutants versus wild type (Supplementary Figure S4).

Finally, given Smaug's major role in AGO1 production and stability in 2-4 hr embryos (reported in the main text), we analyzed the expression of the proteins involved in production of piRNAs (Piwi, AUB, AGO3) and siRNAs (AGO2). We found no significant effect of *smaug* mutations on the expression of these proteins (Supplementary Figure S5).

References

Brennecke, J., A. Aravin, A. Stark, M. Dus, M. Kellis *et al.*, 2007 Discrete small RNA-generating loci as master regulators of transposon activity in Drosophila. Cell 128: 1089-1103.

Brennecke, J., C. Malone, A. Aravin, R. Sachidanandam, A. Stark *et al.*, 2008 An epigenetic role for maternally inherited piRNAs in transposon silencing. Science 322: 1387-1392.

Celniker, S., D. Wheeler, B. Kronmiller, J. Carlson, A. Halpern *et al.*, 2002 Finishing a whole-genome shotgun: release 3 of the Drosophila melanogaster euchromatic genome sequence. Genome Biol 3: RESEARCH0079.

Czech, B., C. D. Malone, R. Zhou, A. Stark, C. Schlingeheyde *et al.*, 2008 An endogenous small interfering RNA pathway in Drosophila. Nature 453: 798-802.

Gunawardane, L., K. Saito, K. Nishida, K. Miyoshi, Y. Kawamura *et al.*, 2007 A slicer-mediated mechanism for repeat-associated siRNA 5' end formation in Drosophila. Science 16: 1587-1590.

Jurka, J., V. V. Kapitonov, A. Pavlicek, P. Klonowski, O. Kohany *et al.*, 2005 Repbase Update, a database of eukaryotic repetitive elements. Cytogenet Genome Res 110: 462-467.

Kozomara, A., and S. Griffiths-Jones, 2011 miRBase: integrating microRNA annotation and deep-sequencing data. Nucleic Acids Res 39: D152-157.

Lau, N. C., N. Robine, R. Martin, W. J. Chung, Y. Niki *et al.*, 2009 Abundant primary piRNAs, endo-siRNAs, and microRNAs in a Drosophila ovary cell line. Genome Res 19: 1776-1785.

Li, C., V. V. Vagin, S. Lee, J. Xu, S. Ma *et al.*, 2009a Collapse of germline piRNAs in the absence of Argonaute3 reveals somatic piRNAs in flies. Cell 137: 509-521.

Li, H., B. Handsaker, A. Wysoker, T. Fennell, J. Ruan *et al.*, 2009b The Sequence Alignment/Map format and SAMtools. Bioinformatics 25: 2078-2079.

Malone, C. D., J. Brennecke, M. Dus, A. Stark, W. R. McCombie *et al.*, 2009 Specialized piRNA pathways act in germline and somatic tissues of the Drosophila ovary. Cell 137: 522-535.

Miyoshi, K., H. Tsukumo, T. Nagami, H. Siomi and M. C. Siomi, 2005 Slicer function of Drosophila Argonautes and its involvement in RISC formation. Genes Dev 19: 2837-2848.

Nishida, K. M., K. Saito, T. Mori, Y. Kawamura, T. Nagami-Okada *et al.*, 2007 Gene silencing mechanisms mediated by Aubergine piRNA complexes in Drosophila male gonad. RNA 13: 1911-1922.

Okamura, K., S. Balla, R. Martin, N. Liu and E. C. Lai, 2008 Two distinct mechanisms generate endogenous siRNAs from bidirectional transcription in Drosophila melanogaster. Nat Struct Mol Biol 15: 581-590.

Quinlan, A. R., and I. M. Hall, 2010 BEDTools: a flexible suite of utilities for comparing genomic features. Bioinformatics 26: 841-842.
