# Supplementary figures and images for: The Smaug RNA-Binding Protein Is Essential for microRNA Synthesis During the Drosophila Maternal-to-Zygotic Transition
Source: G3 (Bethesda). 2016 Sep 1;6(11):3541–51. doi: 10.1534/g3.116.034199 (PMC5100853; doi:10.1534/g3.116.034199)

# Supplementary Figure S1

**A**

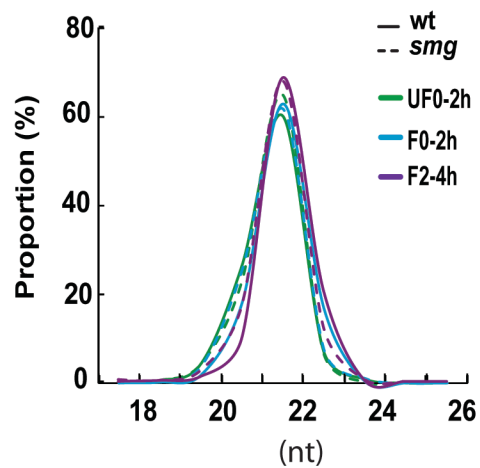

**B**

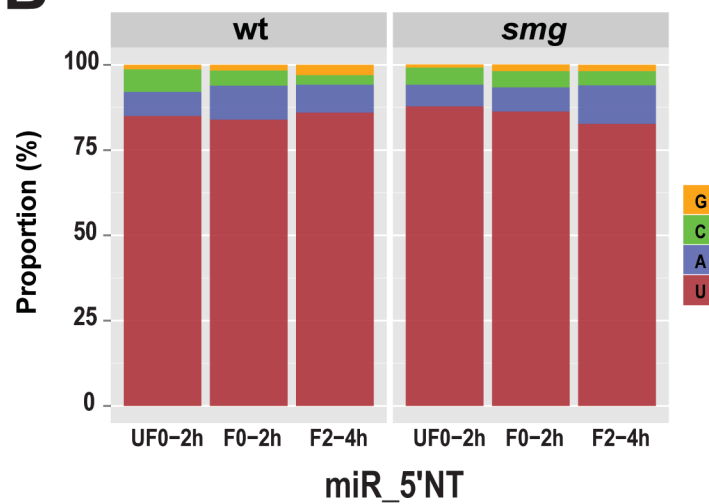

**C**

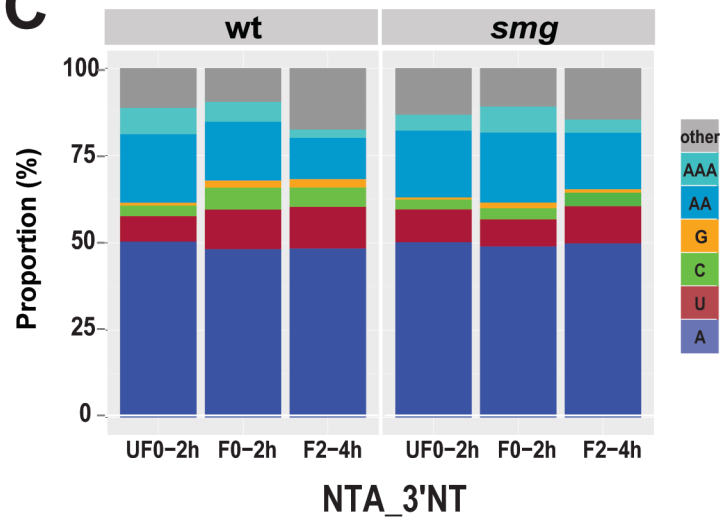

Supplement: Supplemental Material [file supp_g3.116.034199_FigureS1.pdf]

# Supplementary Figure S2

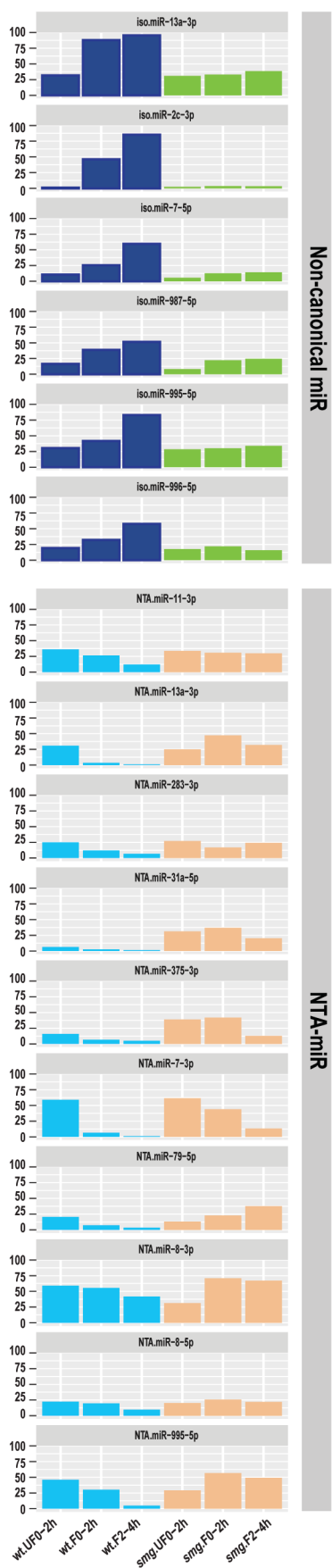

Supplement: Supplemental Material [file supp_g3.116.034199_FigureS2.pdf]

# Supplementary Figure S3

**A**

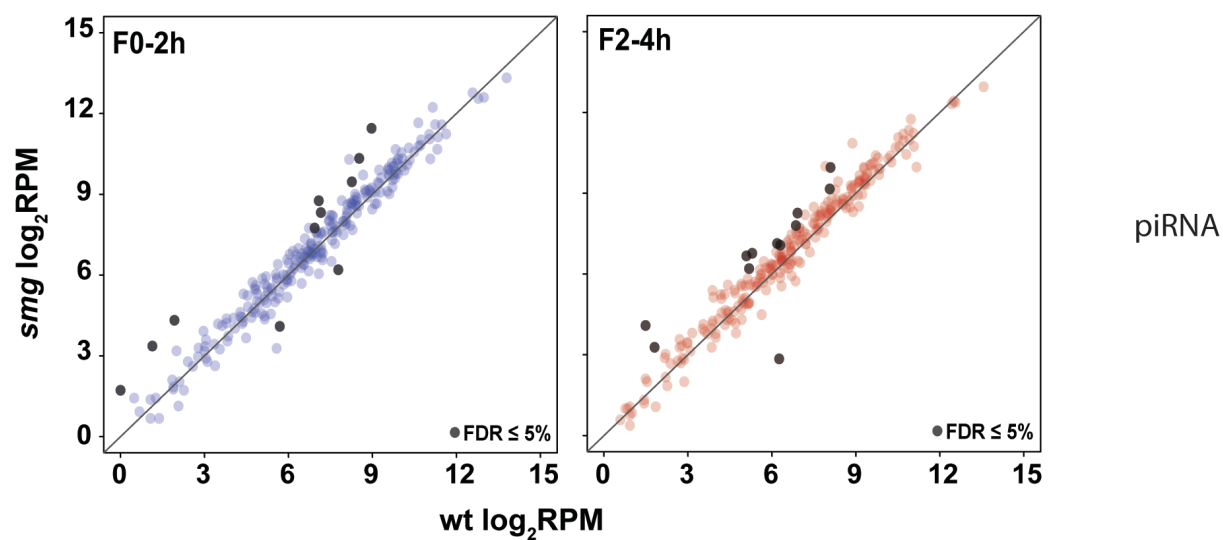

**B**

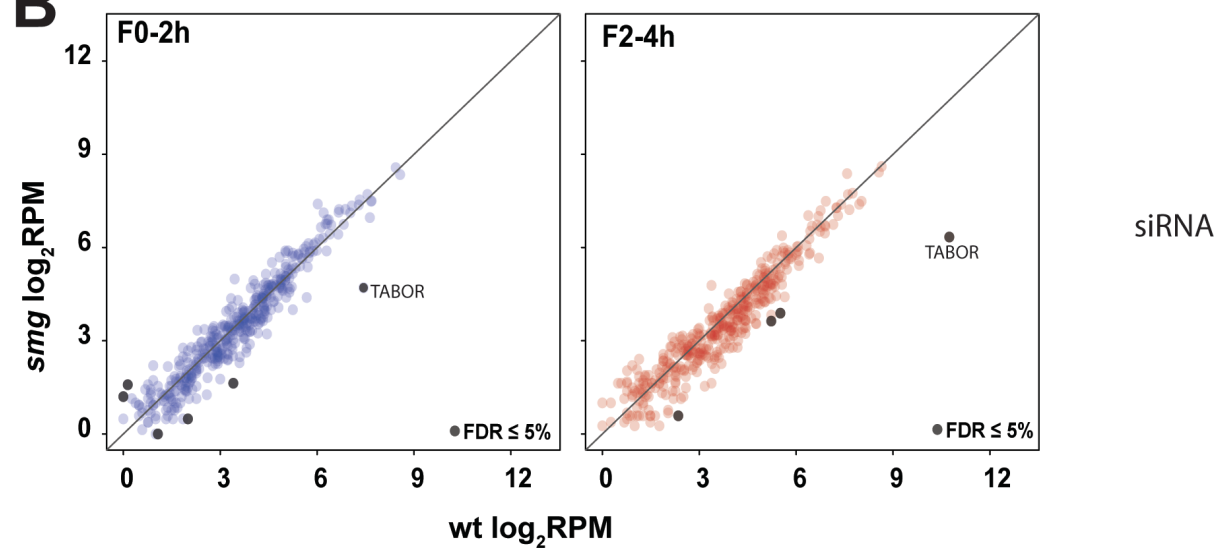

**C**

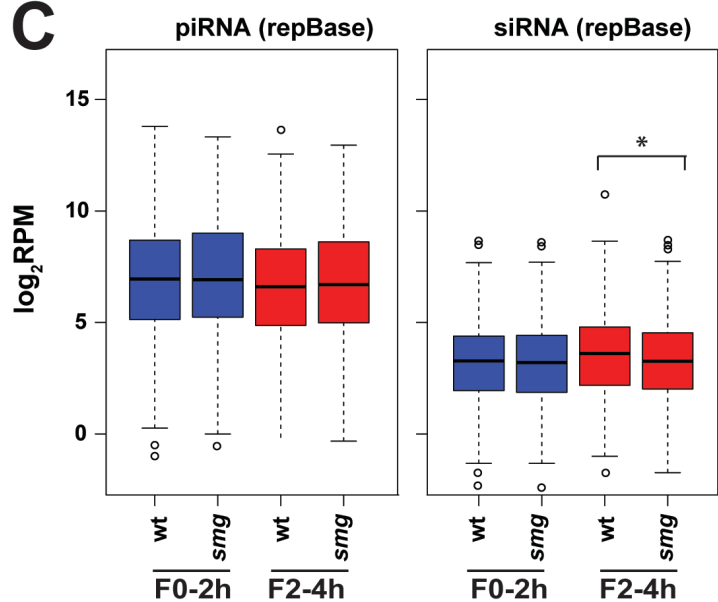

**D**

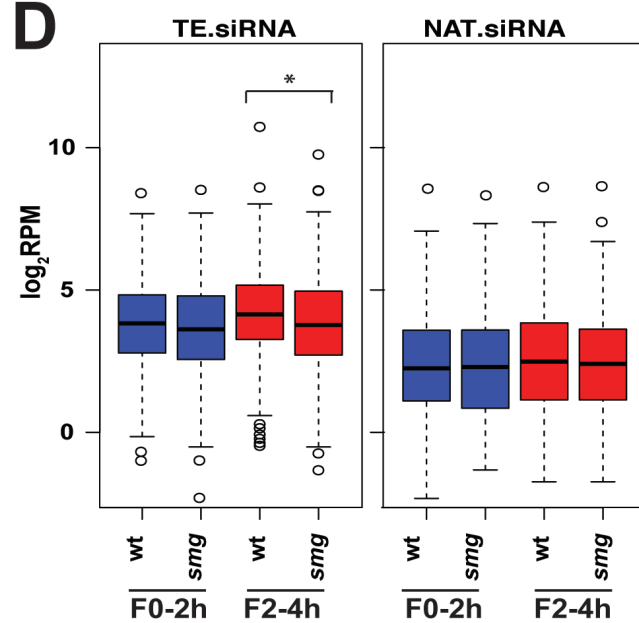

Supplement: Supplemental Material [file supp_g3.116.034199_FigureS3.pdf]

## Supplementary Figure S4

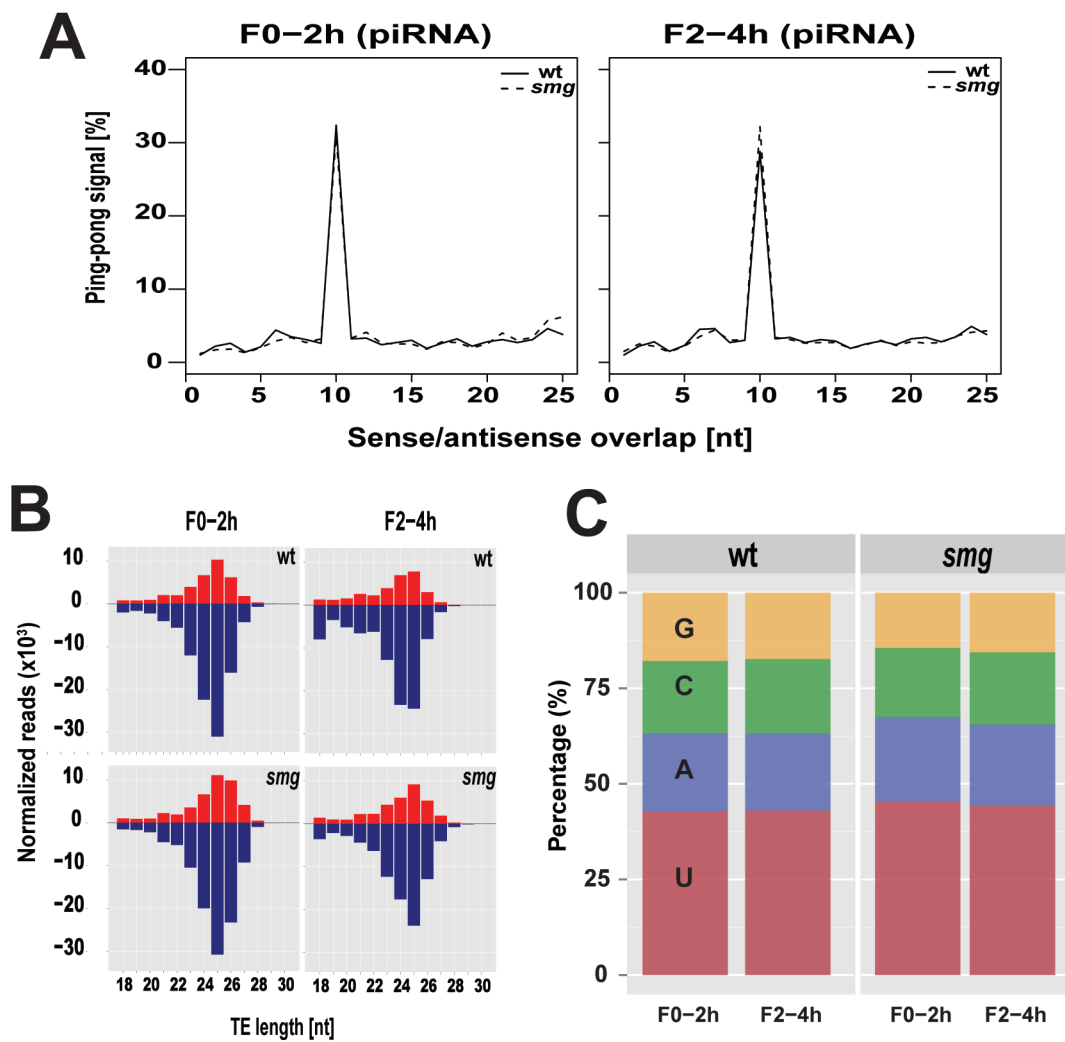

Supplement: Supplemental Material [file supp_g3.116.034199_FigureS4.pdf]

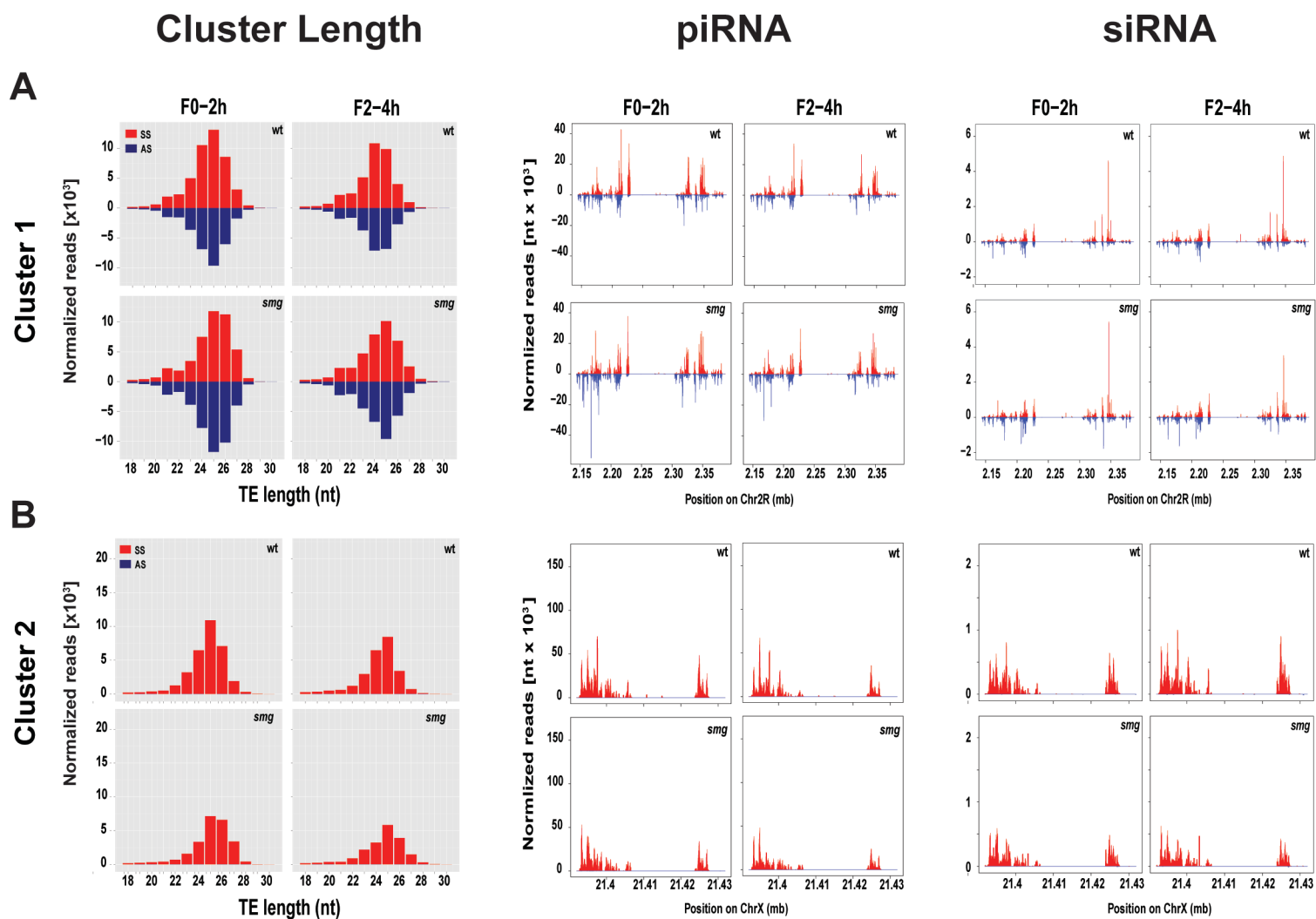

Supplementary Figure S5

Supplement: Supplemental Material [file supp_g3.116.034199_FigureS5.pdf]

# Supplementary Figure S6

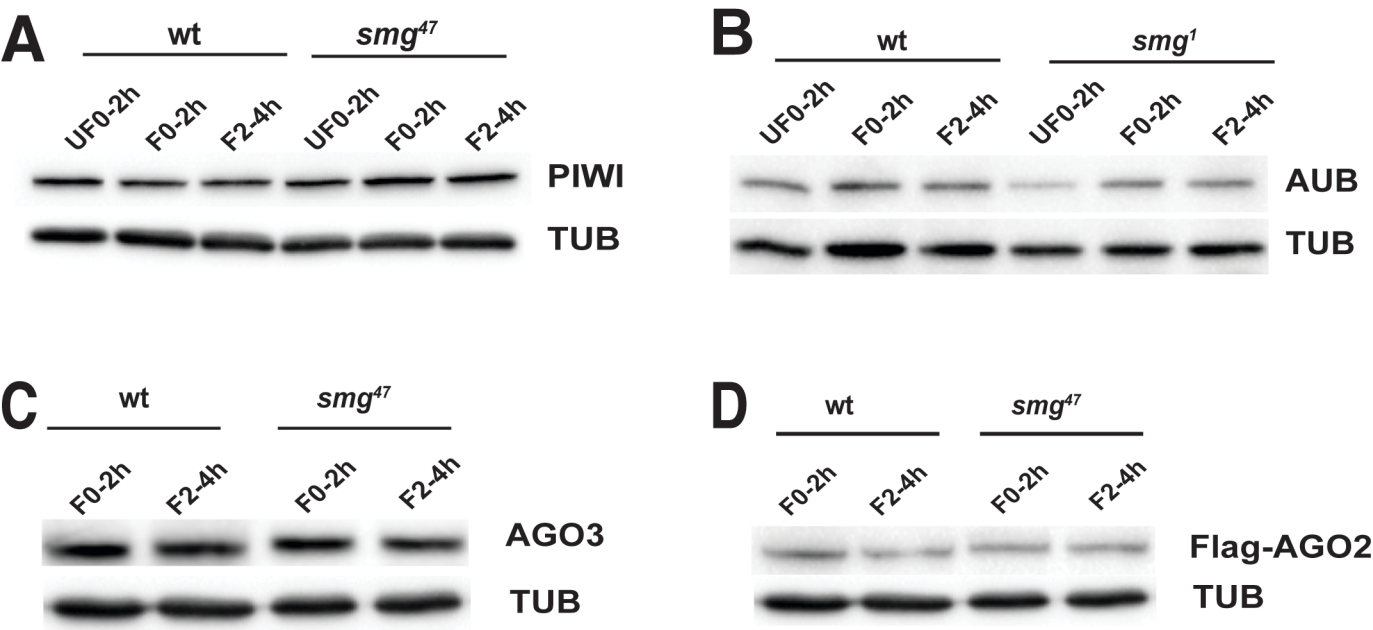

Supplement: Supplemental Material [file supp_g3.116.034199_FigureS6.pdf]
